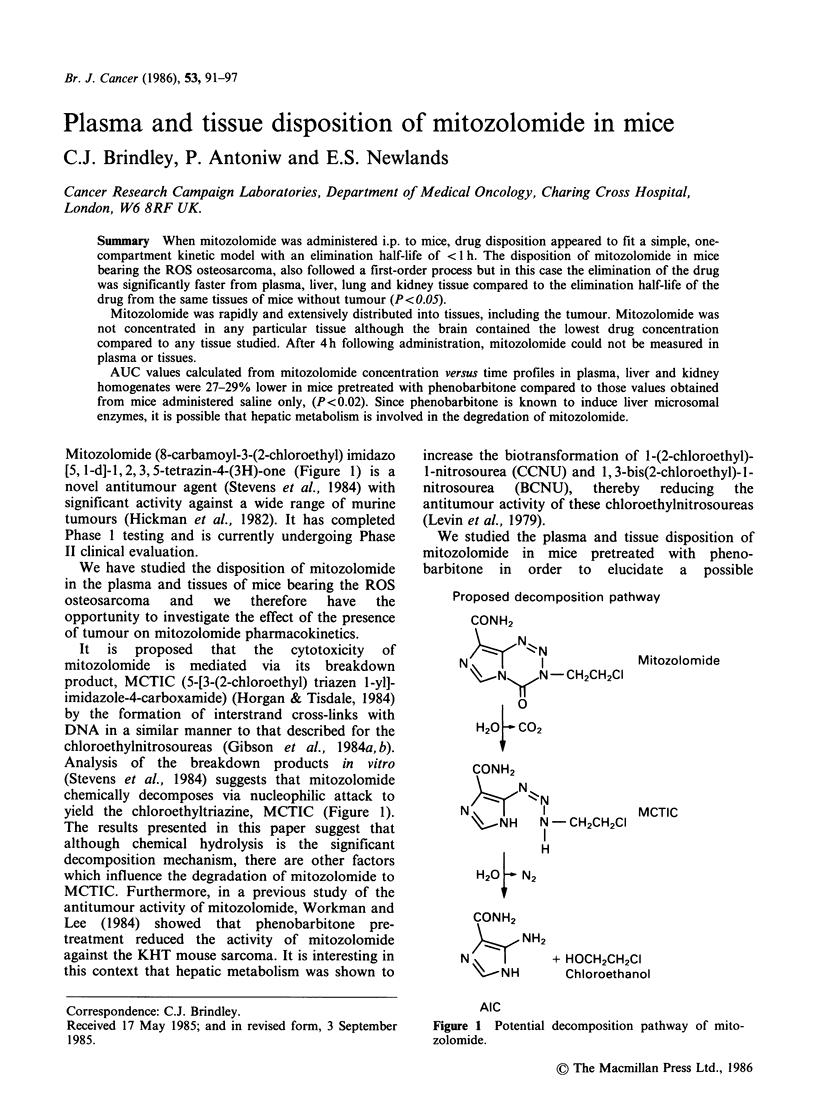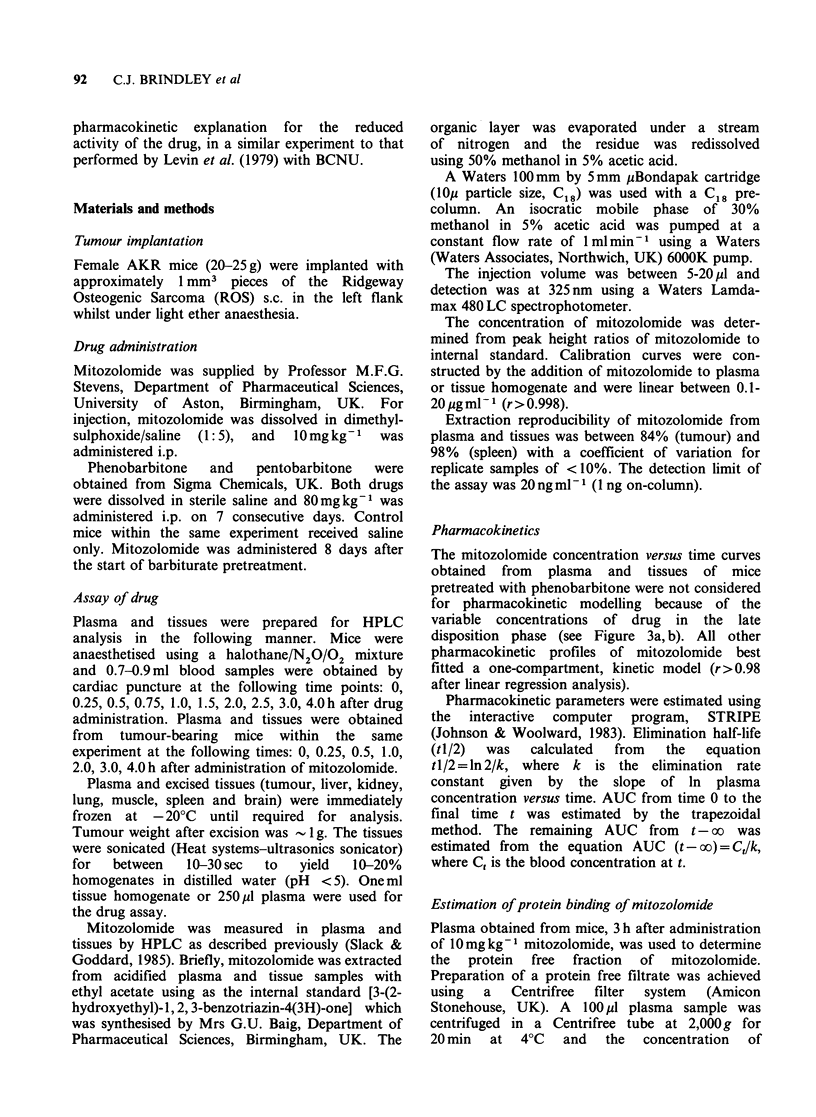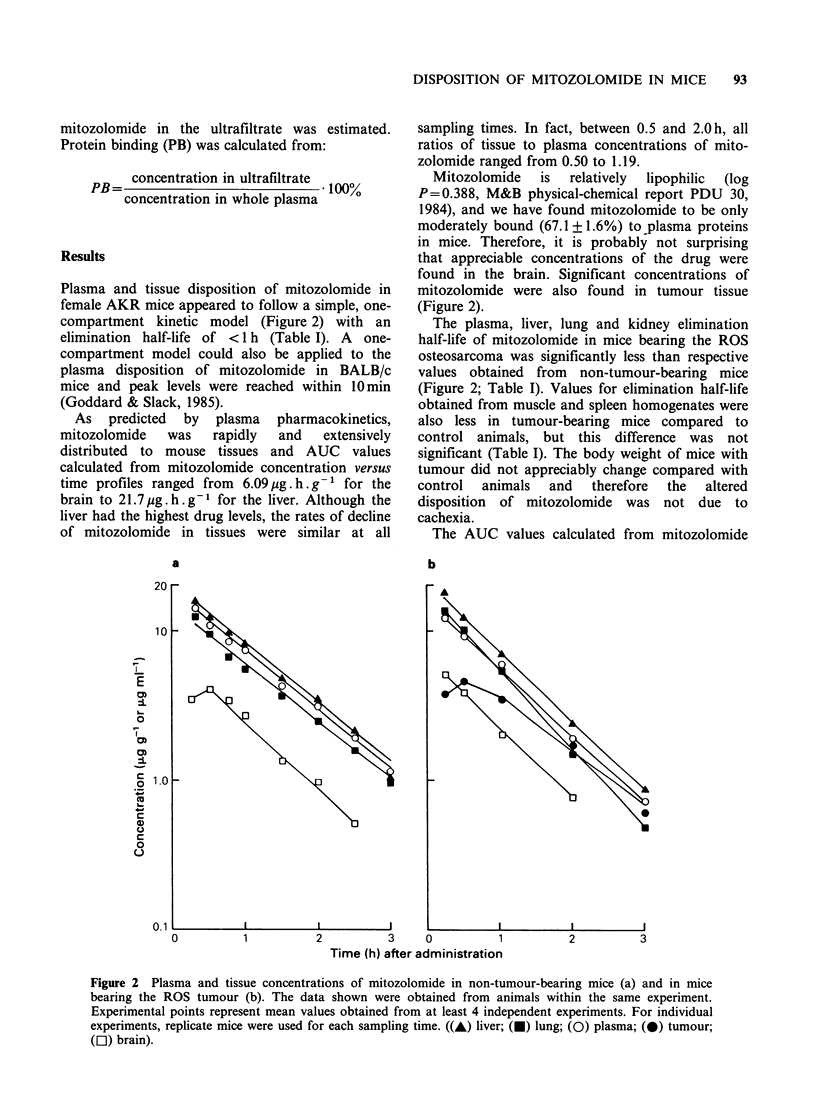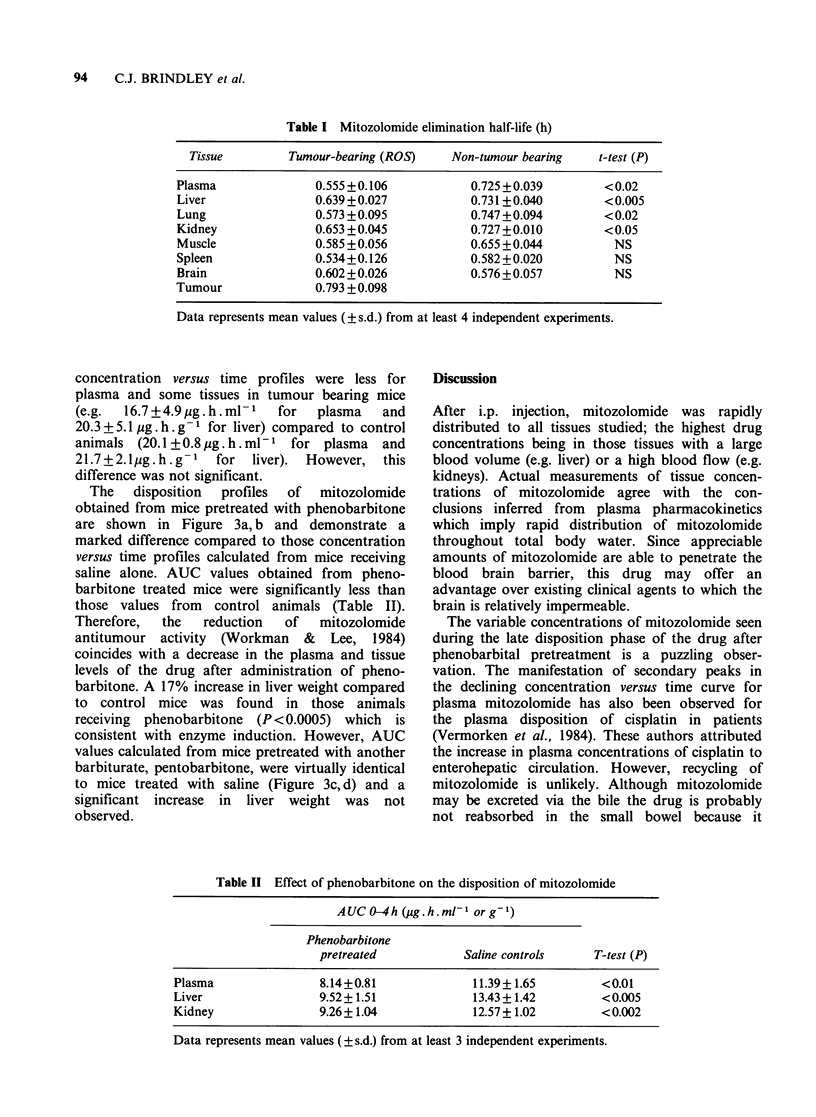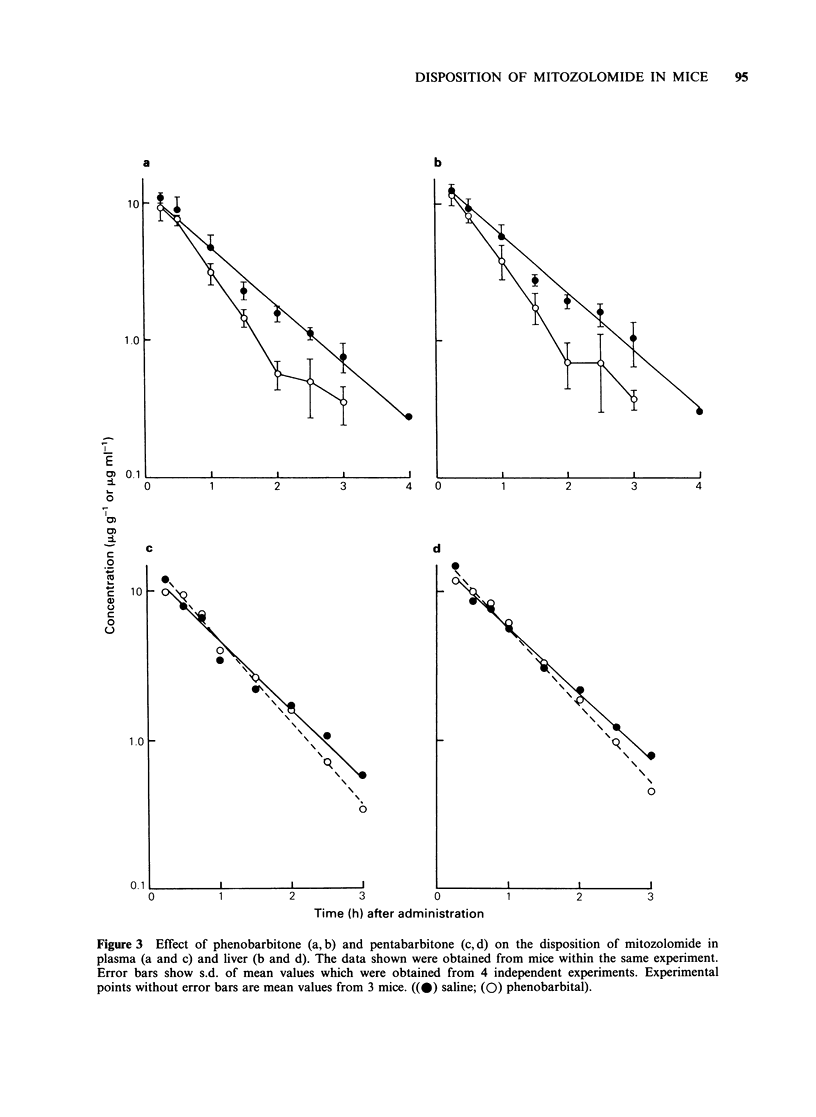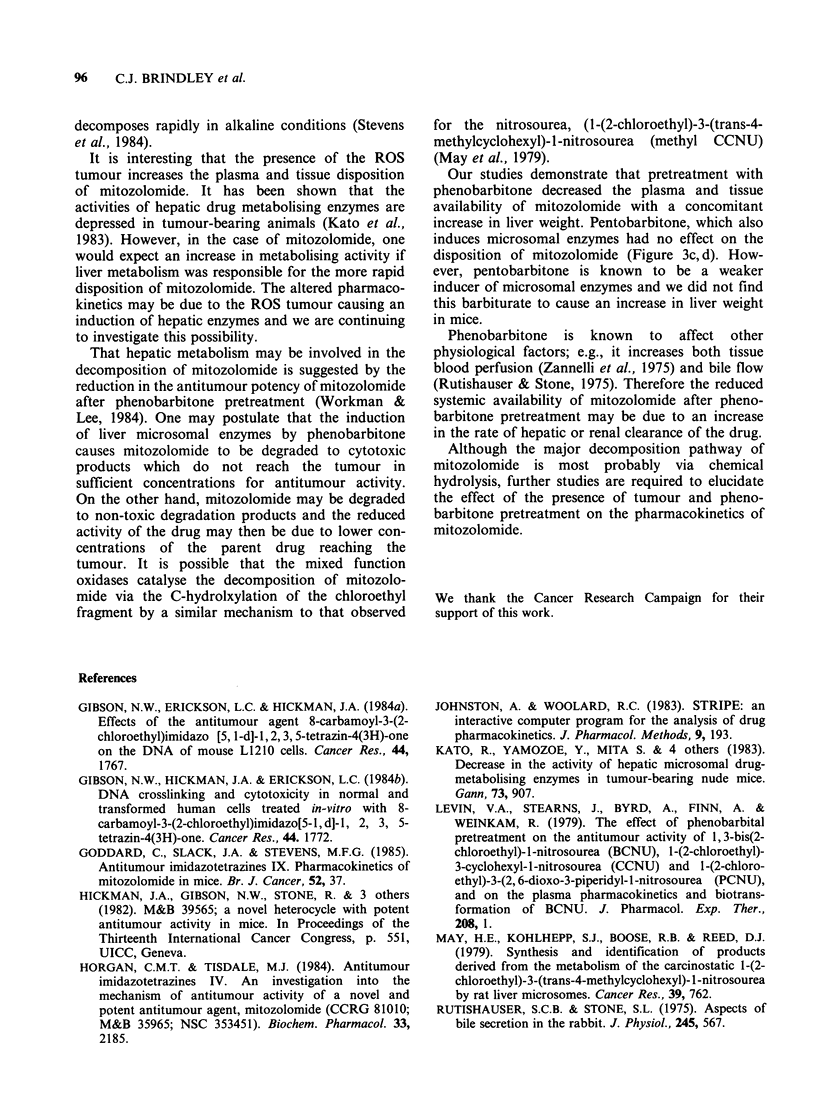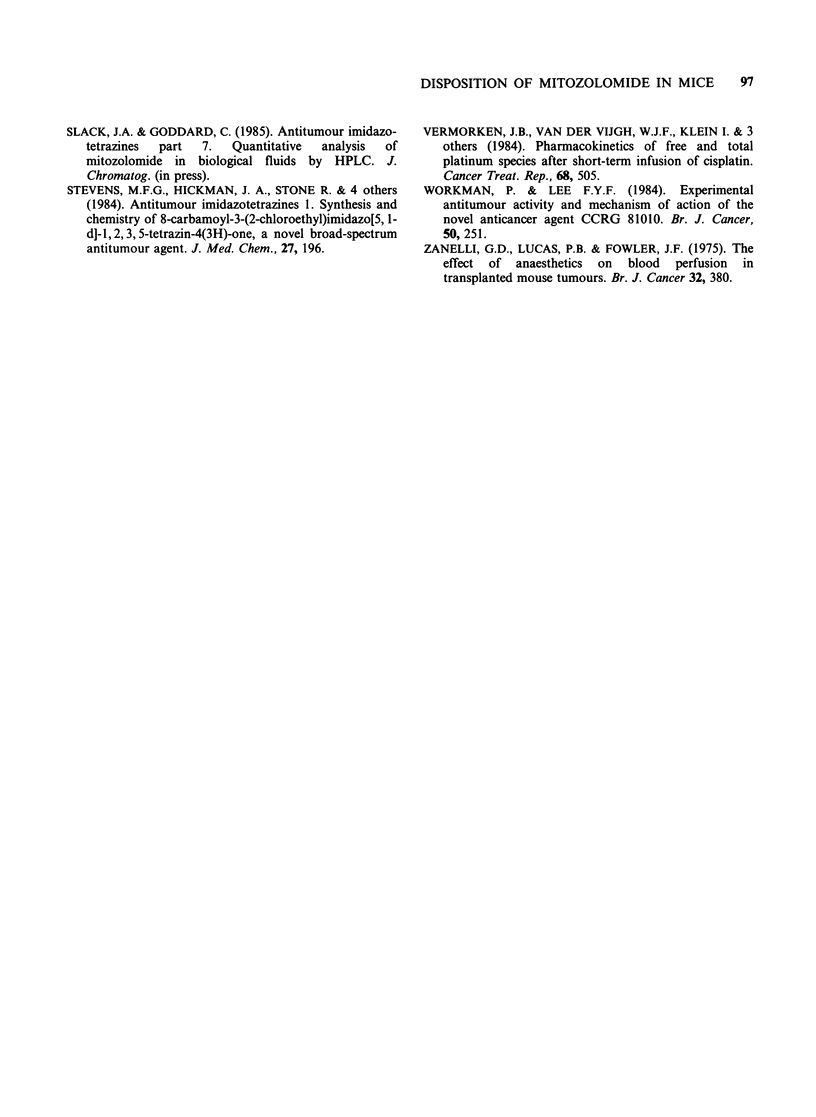# Plasma and tissue disposition of mitozolomide in mice.

**DOI:** 10.1038/bjc.1986.13

**Published:** 1986-01

**Authors:** C. J. Brindley, P. Antoniw, E. S. Newlands

## Abstract

When mitozolomide was administered i.p. to mice, drug disposition appeared to fit a simple, one-compartment kinetic model with an elimination half-life of less than 1 h. The disposition of mitozolomide in mice bearing the ROS osteosarcoma, also followed a first-order process but in this case the elimination of the drug was significantly faster from plasma, liver, lung and kidney tissue compared to the elimination half-life of the drug from the same tissues of mice without tumour (P less than 0.05). Mitozolomide was rapidly and extensively distributed into tissues, including the tumour. Mitozolomide was not concentrated in any particular tissue although the brain contained the lowest drug concentration compared to any tissue studied. After 4 h following administration, mitozolomide could not be measured in plasma or tissues. AUC values calculated from mitozolomide concentration versus time profiles in plasma, liver and kidney homogenates were 27-29% lower in mice pretreated with phenobarbitone compared to those values obtained from mice administered saline only, (P less than 0.02). Since phenobarbitone is known to induce liver microsomal enzymes, it is possible that hepatic metabolism is involved in the degredation of mitozolomide.


*Br. J. Cancer* (1986), **53**, 91–97

### Plasma and tissue disposition of mitozolomide in mice

C.J. Brindley, P. Antoniw and E.S. Newlands

*Cancer Research Campaign Laboratories, Department of Medical Oncology, Charing Cross Hospital, London, W6 8RF UK.*

**Summary** When mitozolomide was administered i.p. to mice, drug disposition appeared to fit a simple, one-compartment kinetic model with an elimination half-life of <1 h. The disposition of mitozolomide in mice bearing the ROS osteosarcoma, also followed a first-order process but in this case the elimination of the drug was significantly faster from plasma, liver, lung and kidney tissue compared to the elimination half-life of the drug from the same tissues of mice without tumour ($P<0.05$).

Mitozolomide was rapidly and extensively distributed into tissues, including the tumour. Mitozolomide was not concentrated in any particular tissue although the brain contained the lowest drug concentration compared to any tissue studied. After 4 h following administration, mitozolomide could not be measured in plasma or tissues.

AUC values calculated from mitozolomide concentration *versus* time profiles in plasma, liver and kidney homogenates were 27–29% lower in mice pretreated with phenobarbitone compared to those values obtained from mice administered saline only, ($P<0.02$). Since phenobarbitone is known to induce liver microsomal enzymes, it is possible that hepatic metabolism is involved in the degradation of mitozolomide.

Mitozolomide (8-carbamoyl-3-(2-chloroethyl) imidazo [5, 1-d]-1, 2, 3, 5-tetrazin-4-(3H)-one (Figure 1) is a novel antitumour agent (Stevens *et al.,* 1984) with significant activity against a wide range of murine tumours (Hickman *et al.,* 1982). It has completed Phase 1 testing and is currently undergoing Phase II clinical evaluation.

We have studied the disposition of mitozolomide in the plasma and tissues of mice bearing the ROS osteosarcoma and we therefore have the opportunity to investigate the effect of the presence of tumour on mitozolomide pharmacokinetics.

It is proposed that the cytotoxicity of mitozolomide is mediated via its breakdown product, MCTIC (5-[3-(2-chloroethyl) triazen 1-yl]-imidazole-4-carboxamide) (Horgan & Tisdale, 1984) by the formation of interstrand cross-links with DNA in a similar manner to that described for the chloroethylnitrosoureas (Gibson *et al.,* 1984*a,b*). Analysis of the breakdown products *in vitro* (Stevens *et al.,* 1984) suggests that mitozolomide chemically decomposes via nucleophilic attack to yield the chloroethyltriazine, MCTIC (Figure 1). The results presented in this paper suggest that although chemical hydrolysis is the significant decomposition mechanism, there are other factors which influence the degradation of mitozolomide to MCTIC. Furthermore, in a previous study of the antitumour activity of mitozolomide, Workman and Lee (1984) showed that phenobarbitone pre-treatment reduced the activity of mitozolomide against the KHT mouse sarcoma. It is interesting in this context that hepatic metabolism was shown to increase the biotransformation of 1-(2-chloroethyl)-1-nitrosourea (CCNU) and 1, 3-bis(2-chloroethyl)-1-nitrosourea (BCNU), thereby reducing the antitumour activity of these chloroethylnitrosoureas (Levin *et al.,* 1979).

We studied the plasma and tissue disposition of mitozolomide in mice pretreated with phenobarbitone in order to elucidate a possible

**Figure 1** Potential decomposition pathway of mitozolomide.

Correspondence: C.J. Brindley.
Received 17 May 1985; and in revised form, 3 September 1985.

© The Macmillan Press Ltd., 1986

pharmacokinetic explanation for the reduced activity of the drug, in a similar experiment to that performed by Levin *et al.* (1979) with BCNU.

### Materials and methods

### *Tumour implantation*

Female AKR mice (20–25 g) were implanted with approximately 1 $mm^3$ pieces of the Ridgeway Osteogenic Sarcoma (ROS) s.c. in the left flank whilst under light ether anaesthesia.

### *Drug administration*

Mitozolomide was supplied by Professor M.F.G. Stevens, Department of Pharmaceutical Sciences, University of Aston, Birmingham, UK. For injection, mitozolomide was dissolved in dimethylsulphoxide/saline (1:5), and 10 mg $kg^{-1}$ was administered i.p.

Phenobarbitone and pentobarbitone were obtained from Sigma Chemicals, UK. Both drugs were dissolved in sterile saline and 80 mg $kg^{-1}$ was administered i.p. on 7 consecutive days. Control mice within the same experiment received saline only. Mitozolomide was administered 8 days after the start of barbiturate pretreatment.

### *Assay of drug*

Plasma and tissues were prepared for HPLC analysis in the following manner. Mice were anaesthetised using a halothane/$N_2O/O_2$ mixture and 0.7–0.9 ml blood samples were obtained by cardiac puncture at the following time points: 0, 0.25, 0.5, 0.75, 1.0, 1.5, 2.0, 2.5, 3.0, 4.0 h after drug administration. Plasma and tissues were obtained from tumour-bearing mice within the same experiment at the following times: 0, 0.25, 0.5, 1.0, 2.0, 3.0, 4.0 h after administration of mitozolomide.

Plasma and excised tissues (tumour, liver, kidney, lung, muscle, spleen and brain) were immediately frozen at −20°C until required for analysis. Tumour weight after excision was ~1 g. The tissues were sonicated (Heat systems–ultrasonics sonicator) for between 10–30 sec to yield 10–20% homogenates in distilled water (pH <5). One ml tissue homogenate or 250 $\mu$l plasma were used for the drug assay.

Mitozolomide was measured in plasma and tissues by HPLC as described previously (Slack & Goddard, 1985). Briefly, mitozolomide was extracted from acidified plasma and tissue samples with ethyl acetate using as the internal standard [3-(2-hydroxyethyl)-1,2,3-benzotriazin-4(3H)-one] which was synthesised by Mrs G.U. Baig, Department of Pharmaceutical Sciences, Birmingham, UK. The organic layer was evaporated under a stream of nitrogen and the residue was redissolved using 50% methanol in 5% acetic acid.

A Waters 100 mm by 5 mm $\mu$Bondapak cartridge (10$\mu$ particle size, $C_{18}$) was used with a $C_{18}$ precolumn. An isocratic mobile phase of 30% methanol in 5% acetic acid was pumped at a constant flow rate of 1 ml $min^{-1}$ using a Waters (Waters Associates, Northwich, UK) 6000K pump.

The injection volume was between 5-20 $\mu$l and detection was at 325 nm using a Waters Lamdamax 480 LC spectrophotometer.

The concentration of mitozolomide was determined from peak height ratios of mitozolomide to internal standard. Calibration curves were constructed by the addition of mitozolomide to plasma or tissue homogenate and were linear between 0.1-20 $\mu$g $ml^{-1}$ ($r>0.998$).

Extraction reproducibility of mitozolomide from plasma and tissues was between 84% (tumour) and 98% (spleen) with a coefficient of variation for replicate samples of <10%. The detection limit of the assay was 20 ng $ml^{-1}$ (1 ng on-column).

### *Pharmacokinetics*

The mitozolomide concentration *versus* time curves obtained from plasma and tissues of mice pretreated with phenobarbitone were not considered for pharmacokinetic modelling because of the variable concentrations of drug in the late disposition phase (see Figure 3a, b). All other pharmacokinetic profiles of mitozolomide best fitted a one-compartment, kinetic model ($r>0.98$ after linear regression analysis).

Pharmacokinetic parameters were estimated using the interactive computer program, STRIPE (Johnson & Woolward, 1983). Elimination half-life ($t1/2$) was calculated from the equation $t1/2 = \ln 2/k$, where $k$ is the elimination rate constant given by the slope of ln plasma concentration *versus* time. AUC from time 0 to the final time $t$ was estimated by the trapezoidal method. The remaining AUC from $t-\infty$ was estimated from the equation AUC $(t-\infty) = C_t/k$, where $C_t$ is the blood concentration at $t$.

### *Estimation of protein binding of mitozolomide*

Plasma obtained from mice, 3 h after administration of 10 mg $kg^{-1}$ mitozolomide, was used to determine the protein free fraction of mitozolomide. Preparation of a protein free filtrate was achieved using a Centrifree filter system (Amicon Stonehouse, UK). A 100 $\mu$l plasma sample was centrifuged in a Centrifree tube at 2,000 $g$ for 20 min at 4°C and the concentration of mitozolomide in the ultrafiltrate was estimated. Protein binding (PB) was calculated from:

$$PB=\frac{\text{concentration in ultrafiltrate}}{\text{concentration in whole plasma}}\cdot 100\%$$

### Results

Plasma and tissue disposition of mitozolomide in female AKR mice appeared to follow a simple, one-compartment kinetic model (Figure 2) with an elimination half-life of <1 h (Table I). A one-compartment model could also be applied to the plasma disposition of mitozolomide in BALB/c mice and peak levels were reached within 10 min (Goddard & Slack, 1985).

As predicted by plasma pharmacokinetics, mitozolomide was rapidly and extensively distributed to mouse tissues and AUC values calculated from mitozolomide concentration *versus* time profiles ranged from 6.09 $\mu$g.h.$g^{-1}$ for the brain to 21.7 $\mu$g.h.$g^{-1}$ for the liver. Although the liver had the highest drug levels, the rates of decline of mitozolomide in tissues were similar at all sampling times. In fact, between 0.5 and 2.0 h, all ratios of tissue to plasma concentrations of mitozolomide ranged from 0.50 to 1.19.

Mitozolomide is relatively lipophilic (log $P=0.388$, M&B physical-chemical report PDU 30, 1984), and we have found mitozolomide to be only moderately bound (67.1 ± 1.6%) to plasma proteins in mice. Therefore, it is probably not surprising that appreciable concentrations of the drug were found in the brain. Significant concentrations of mitozolomide were also found in tumour tissue (Figure 2).

The plasma, liver, lung and kidney elimination half-life of mitozolomide in mice bearing the ROS osteosarcoma was significantly less than respective values obtained from non-tumour-bearing mice (Figure 2; Table I). Values for elimination half-life obtained from muscle and spleen homogenates were also less in tumour-bearing mice compared to control animals, but this difference was not significant (Table I). The body weight of mice with tumour did not appreciably change compared with control animals and therefore the altered disposition of mitozolomide was not due to cachexia.

The AUC values calculated from mitozolomide

**Figure 2** Plasma and tissue concentrations of mitozolomide in non-tumour-bearing mice (a) and in mice bearing the ROS tumour (b). The data shown were obtained from animals within the same experiment. Experimental points represent mean values obtained from at least 4 independent experiments. For individual experiments, replicate mice were used for each sampling time. ((▲) liver; (■) lung; (○) plasma; (●) tumour; (□) brain).

**Table I** Mitozolomide elimination half-life (h)

| *Tissue* | *Tumour-bearing (ROS)* | *Non-tumour bearing* | *t-test (P)* |
|---|---|---|---|
| Plasma | 0.555 ± 0.106 | 0.725 ± 0.039 | <0.02 |
| Liver | 0.639 ± 0.027 | 0.731 ± 0.040 | <0.005 |
| Lung | 0.573 ± 0.095 | 0.747 ± 0.094 | <0.02 |
| Kidney | 0.653 ± 0.045 | 0.727 ± 0.010 | <0.05 |
| Muscle | 0.585 ± 0.056 | 0.655 ± 0.044 | NS |
| Spleen | 0.534 ± 0.126 | 0.582 ± 0.020 | NS |
| Brain | 0.602 ± 0.026 | 0.576 ± 0.057 | NS |
| Tumour | 0.793 ± 0.098 | | |

Data represents mean values (±s.d.) from at least 4 independent experiments.

concentration *versus* time profiles were less for plasma and some tissues in tumour bearing mice (e.g. 16.7 ± 4.9 $\mu$g.h.ml$^{-1}$ for plasma and 20.3 ± 5.1 $\mu$g.h.g$^{-1}$ for liver) compared to control animals (20.1 ± 0.8 $\mu$g.h.ml$^{-1}$ for plasma and 21.7 ± 2.1 $\mu$g.h.g$^{-1}$ for liver). However, this difference was not significant.

The disposition profiles of mitozolomide obtained from mice pretreated with phenobarbitone are shown in Figure 3a, b and demonstrate a marked difference compared to those concentration *versus* time profiles calculated from mice receiving saline alone. AUC values obtained from phenobarbitone treated mice were significantly less than those values from control animals (Table II). Therefore, the reduction of mitozolomide antitumour activity (Workman & Lee, 1984) coincides with a decrease in the plasma and tissue levels of the drug after administration of phenobarbitone. A 17% increase in liver weight compared to control mice was found in those animals receiving phenobarbitone ($P<0.0005$) which is consistent with enzyme induction. However, AUC values calculated from mice pretreated with another barbiturate, pentobarbitone, were virtually identical to mice treated with saline (Figure 3c, d) and a significant increase in liver weight was not observed.

### Discussion

After i.p. injection, mitozolomide was rapidly distributed to all tissues studied; the highest drug concentrations being in those tissues with a large blood volume (e.g. liver) or a high blood flow (e.g. kidneys). Actual measurements of tissue concentrations of mitozolomide agree with the conclusions inferred from plasma pharmacokinetics which imply rapid distribution of mitozolomide throughout total body water. Since appreciable amounts of mitozolomide are able to penetrate the blood brain barrier, this drug may offer an advantage over existing clinical agents to which the brain is relatively impermeable.

The variable concentrations of mitozolomide seen during the late disposition phase of the drug after phenobarbital pretreatment is a puzzling observation. The manifestation of secondary peaks in the declining concentration *versus* time curve for plasma mitozolomide has also been observed for the plasma disposition of cisplatin in patients (Vermorken *et al.*, 1984). These authors attributed the increase in plasma concentrations of cisplatin to enterohepatic circulation. However, recycling of mitozolomide is unlikely. Although mitozolomide may be excreted via the bile the drug is probably not reabsorbed in the small bowel because it

**Table II** Effect of phenobarbitone on the disposition of mitozolomide

| | *AUC 0–4 h* ($\mu$*g.h.ml*$^{-1}$ *or g*$^{-1}$) | | |
|---|---|---|---|
| | *Phenobarbitone pretreated* | *Saline controls* | *T-test (P)* |
| Plasma | 8.14 ± 0.81 | 11.39 ± 1.65 | <0.01 |
| Liver | 9.52 ± 1.51 | 13.43 ± 1.42 | <0.005 |
| Kidney | 9.26 ± 1.04 | 12.57 ± 1.02 | <0.002 |

Data represents mean values (±s.d.) from at least 3 independent experiments.

**Figure 3** Effect of phenobarbitone (a, b) and pentabarbitone (c, d) on the disposition of mitozolomide in plasma (a and c) and liver (b and d). The data shown were obtained from mice within the same experiment. Error bars show s.d. of mean values which were obtained from 4 independent experiments. Experimental points without error bars are mean values from 3 mice. ((●) saline; (○) phenobarbital).

decomposes rapidly in alkaline conditions (Stevens *et al.*, 1984).

It is interesting that the presence of the ROS tumour increases the plasma and tissue disposition of mitozolomide. It has been shown that the activities of hepatic drug metabolising enzymes are depressed in tumour-bearing animals (Kato *et al.*, 1983). However, in the case of mitozolomide, one would expect an increase in metabolising activity if liver metabolism was responsible for the more rapid disposition of mitozolomide. The altered pharmacokinetics may be due to the ROS tumour causing an induction of hepatic enzymes and we are continuing to investigate this possibility.

That hepatic metabolism may be involved in the decomposition of mitozolomide is suggested by the reduction in the antitumour potency of mitozolomide after phenobarbitone pretreatment (Workman & Lee, 1984). One may postulate that the induction of liver microsomal enzymes by phenobarbitone causes mitozolomide to be degraded to cytotoxic products which do not reach the tumour in sufficient concentrations for antitumour activity. On the other hand, mitozolomide may be degraded to non-toxic degradation products and the reduced activity of the drug may then be due to lower concentrations of the parent drug reaching the tumour. It is possible that the mixed function oxidases catalyse the decomposition of mitozolomide via the C-hydrolxylation of the chloroethyl fragment by a similar mechanism to that observed for the nitrosourea, (1-(2-chloroethyl)-3-(trans-4-methylcyclohexyl)-1-nitrosourea (methyl CCNU) (May *et al.*, 1979).

Our studies demonstrate that pretreatment with phenobarbitone decreased the plasma and tissue availability of mitozolomide with a concomitant increase in liver weight. Pentobarbitone, which also induces microsomal enzymes had no effect on the disposition of mitozolomide (Figure 3c, d). However, pentobarbitone is known to be a weaker inducer of microsomal enzymes and we did not find this barbiturate to cause an increase in liver weight in mice.

Phenobarbitone is known to affect other physiological factors; e.g., it increases both tissue blood perfusion (Zannelli *et al.*, 1975) and bile flow (Rutishauser & Stone, 1975). Therefore the reduced systemic availability of mitozolomide after phenobarbitone pretreatment may be due to an increase in the rate of hepatic or renal clearance of the drug.

Although the major decomposition pathway of mitozolomide is most probably via chemical hydrolysis, further studies are required to elucidate the effect of the presence of tumour and phenobarbitone pretreatment on the pharmacokinetics of mitozolomide.

We thank the Cancer Research Campaign for their support of this work.

### References

GIBSON, N.W., ERICKSON, L.C. & HICKMAN, J.A. (1984*a*). Effects of the antitumour agent 8-carbamoyl-3-(2-chloroethyl)imidazo [5, 1-d]-1, 2, 3, 5-tetrazin-4(3H)-one on the DNA of mouse L1210 cells. *Cancer Res.*, **44**, 1767.

GIBSON, N.W., HICKMAN, J.A. & ERICKSON, L.C. (1984*b*). DNA crosslinking and cytotoxicity in normal and transformed human cells treated *in-vitro* with 8-carbamoyl-3-(2-chloroethyl)imidazo[5-1, d]-1, 2, 3, 5-tetrazin-4(3H)-one. *Cancer Res.*, **44**. 1772.

GODDARD, C., SLACK, J.A. & STEVENS, M.F.G. (1985). Antitumour imidazotetrazines IX. Pharmacokinetics of mitozolomide in mice. *Br. J. Cancer*, **52**, 37.

HICKMAN, J.A., GIBSON, N.W., STONE, R. & 3 others (1982). M&B 39565; a novel heterocycle with potent antitumour activity in mice. In Proceedings of the Thirteenth International Cancer Congress, p. 551, UICC, Geneva.

HORGAN, C.M.T. & TISDALE, M.J. (1984). Antitumour imidazotetrazines IV. An investigation into the mechanism of antitumour activity of a novel and potent antitumour agent, mitozolomide (CCRG 81010; M&B 35965; NSC 353451). *Biochem. Pharmacol.* **33**, 2185.

JOHNSTON, A. & WOOLARD, R.C. (1983). STRIPE: an interactive computer program for the analysis of drug pharmacokinetics. *J. Pharmacol. Methods*, **9**, 193.

KATO, R., YAMOZOE, Y., MITA S. & 4 others (1983). Decrease in the activity of hepatic microsomal drug-metabolising enzymes in tumour-bearing nude mice. *Gann*, **73**, 907.

LEVIN, V.A., STEARNS, J., BYRD, A., FINN, A. & WEINKAM, R. (1979). The effect of phenobarbital pretreatment on the antitumour activity of 1, 3-bis(2-chloroethyl)-1-nitrosourea (BCNU), 1-(2-chloroethyl)-3-cyclohexyl-1-nitrosourea (CCNU) and 1-(2-chloroethyl)-3-(2, 6-dioxo-3-piperidyl-1-nitrosourea (PCNU), and on the plasma pharmacokinetics and biotransformation of BCNU. *J.* Pharmacol. *Exp. Ther.*, **208**, 1.

MAY, H.E., KOHLHEPP, S.J., BOOSE, R.B. & REED, D.J. (1979). Synthesis and identification of products derived from the metabolism of the carcinostatic 1-(2-chloroethyl)-3-(trans-4-methylcyclohexyl)-1-nitrosourea by rat liver microsomes. *Cancer Res.*, **39**, 762.

RUTISHAUSER, S.C.B. & STONE, S.L. (1975). Aspects of bile secretion in the rabbit. *J. Physiol.*, **245**, 567.

SLACK, J.A. & GODDARD, C. (1985). Antitumour imidazotetrazines part 7. Quantitative analysis of mitozolomide in biological fluids by HPLC. *J. Chromatog.* (in press).

STEVENS, M.F.G., HICKMAN, J. A., STONE R. & 4 others (1984). Antitumour imidazotetrazines 1. Synthesis and chemistry of 8-carbamoyl-3-(2-chloroethyl)imidazo[5, 1-d]-1, 2, 3, 5-tetrazin-4(3H)-one, a novel broad-spectrum antitumour agent. *J. Med. Chem.*, **27**, 196.

VERMORKEN, J.B., VAN DER VIJGH, W.J.F., KLEIN I. & 3 others (1984). Pharmacokinetics of free and total platinum species after short-term infusion of cisplatin. *Cancer Treat. Rep.*, **68**, 505.

WORKMAN, P. & LEE F.Y.F. (1984). Experimental antitumour activity and mechanism of action of the novel anticancer agent CCRG 81010. *Br. J. Cancer*, **50**, 251.

ZANELLI, G.D., LUCAS, P.B. & FOWLER, J.F. (1975). The effect of anaesthetics on blood perfusion in transplanted mouse tumours. *Br. J. Cancer* **32**, 380.